# Dengue and Flavivirus Co-Infections: Challenges in Diagnosis, Treatment, and Disease Management

**DOI:** 10.3390/ijms26146609

**Published:** 2025-07-10

**Authors:** Rosmen Sufi Aiman Sabrina, Nor Azila Muhammad Azami, Wei Boon Yap

**Affiliations:** 1 Center for Toxicology and Health Risk Studies, Faculty of Health Sciences, Universiti Kebangsaan Malaysia, Kuala Lumpur 50300, Malaysia; sabrinarosmen@gmail.com; 2 UKM Medical Molecular Biology Institute, Universiti Kebangsaan Malaysia, Kuala Lumpur 56000, Malaysia; azila_azami@ukm.edu.my; 3 One Health UKM, Universiti Kebangsaan Malaysia, Bangi 43600, Malaysia

**Keywords:** dengue virus, flavivirus co-infection, diagnostic, therapeutics, antivirals

## Abstract

Co-infections of dengue serotypes and dengue with other flaviviruses pose substantial hurdles in disease diagnosis, treatment options, and disease management. The overlapping geographic distributions and mosquito vectors significantly enhance the probability of co-infections. Co-infections may result in more severe disease outcomes due to elevated viral loads, modulation of the immune response, and antibody enhancement. Cross-reactivity in serological assays and the likeness of clinical presentations add to the ongoing challenges in disease diagnosis. Molecular diagnostics such as reverse transcription polymerase chain reaction (RT-PCR) and next-generation sequencing (NGS) are, therefore, employed for more specific disease diagnosis although requiring substantial resources. Despite the advancements, specific anti-flaviviral therapy is still limited, hence the urgency for further investigative research into various therapeutic approaches, including peptide inhibitors, host-targeted therapies, and RNA-based interventions. This review discusses the epidemiology, clinical ramifications, and diagnostic obstacles associated with flavivirus co-infections whilst assessing prospective strategies for better disease prevention, treatment, and management. Addressing these critical gaps is essential for disease mitigation whilst improving patient management especially in regions where co-circulation of flaviviruses is common and their diseases are highly endemic.

## 1. Introduction

Dengue is affecting one third of the world population especially in the subtropical and tropical countries. The disease is transmitted by infected female *Aedes* mosquitoes such as *Aedes albopictus* and *Aedes aegypti* through blood meal. As of 2024, the World Health Organization (WHO) has received more than 12.3 million cases, nearly doubling that of the previous year’s [1]. The causative agent of dengue, the dengue virus (DENV), is a single-stranded, non-segmented RNA virus from the flavivirus genus. The viral positive-strand RNA genome is encased within a capsid, known as nucleocapsid. The nucleocapsid is enveloped and appears in a roughly spherical shape, hence the spherical morphology of DENV [2]. DENV is categorized into four closely related serotypes, which are DENV-1, DENV-2, DENV-3, and DENV-4. They form distinctive interactions with host antibodies, thereby giving rise to their varied antigenicity [3,4].

Given their respective antigenic variations, it is suggested to identify the infecting serotypes in dengue patients because they can produce different clinical outcomes following the infections [5]. For the same reason, in the context of co-infection, it is of utmost importance to identify the serotypes present simultaneously in patients in order to provide more timely and better disease management [6]. Generally, a co-infection refers to a simultaneous infection of two or more different pathogens, which can include various viruses, bacteria, fungi, or parasites in a host [7]. Bosch et al. [8] studied how interactions among DENV serotypes and their seasonal variations impacted dengue transmission and influenced the effectiveness of vector control. The study concluded that interactions among DENV serotypes were rather complicated and, therefore, entail more rigorous investigations to fully delineate the relationship between the infecting agents and the behavior of the disease. Moreover, to better decipher the interactions among DENV serotypes, particularly in co-infections, it warrants long-term data on DENV serotypes in co-infections so one can accurately model and understand the interaction dynamics [9]. This review was, therefore, carried out to discuss the epidemiology, clinical ramifications, and diagnostic obstacles associated with flavivirus co-infections whilst assessing prospective strategies for better disease prevention, treatment, and management. Bridging these critical gaps is essential for effective disease mitigation and improved patient management, particularly in regions where flaviviruses co-circulate and their associated diseases are highly endemic.

## 2. Methods

**Figure 1:**
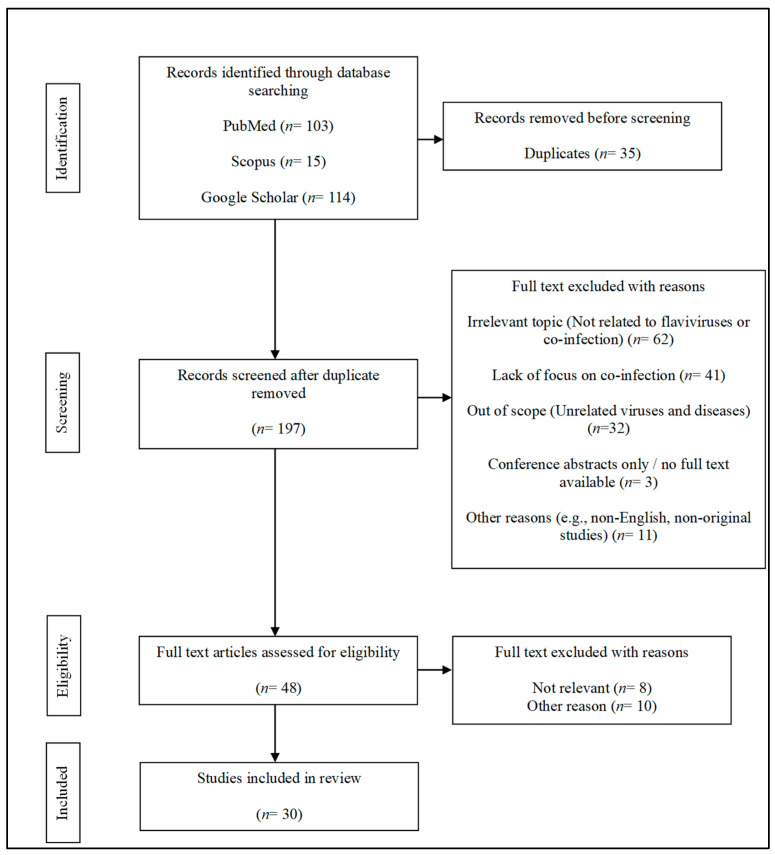
PRISMA flowchart of literature search strategy.

Relevant literature was identified through comprehensive searches of Google Scholar, PubMed, Web of Science (WOS), and Scopus (Figure 1). The search strategy employed combinations of keywords such as “coinfection of flaviviruses” and/or “effect of coinfection” and/or “flavivirus interaction” and/or “future approaches” and/or “diagnosis of flavivirus coinfection”. The search was restricted to English-language articles published between 2002 and 2024, with the inclusion of a limited number of relevant studies from 1990 to 1997. The references from those earlier years were included due to their foundational contributions to the understanding of flavivirus biology and, therefore, were relevant to the scope of the current study.

In brief, a total of 232 articles were shortlisted and then further screened for duplicate, irrelevant, and inconclusive content. At the end of the article screening and selection, 30 articles were finalized for content analysis. Priority was given to recent and original research articles focusing on co-infections between flaviviruses such as DENV, yellow fever virus (YFV), Zika virus (ZIKV), and West Nile virus (WNV). Studies focusing on the detection of flaviviruses in coinfection contexts were also included to enhance diagnostic and methodological insights. Figure 1 depicts the flow of article selection and screening.

## 3. Co-Infection: Occurrence and Clinical Relevance

In regions where multiple DENV serotypes are concurrently transmitted, clinical cases caused by more than one serotype may be more prevalent than previously recognized [10]. In endemic or hyperendemic areas, it offers greater opportunities for mosquitoes to acquire two or more DENV serotypes simultaneously [11]. This is partly explained by vector competence, which is shaped by a complex interplay of biological and ecological factors, including viral interactions within the vector, mosquito immune responses, and feeding behavior [12]. In a co-infection, the viruses may interact synergistically or competitively in the mosquito’s midgut, affecting the virus’s capacity to replicate, disseminate, and eventually spread [13].

Simultaneous infections by multiple serotypes are expected to severely impact the clinical manifestations of dengue. When two DENV serotypes coexist within an individual, they might elevate the overall DENV viremia level, infect high numbers of leukocytes, and potentially induce substantial cytotoxicity due to the culminating cytokine storm [14], ultimately leading to severe symptoms and disease emergence [15]. This was evident in previously described dengue co-infections, where a strong correlation with severe clinical disease, including dengue hemorrhagic fever (DHF) and dengue shock syndrome (DSS), was documented, possibly due to markedly heightened immune response and increased viremia [16,17]. Although some studies reported no significant differences in disease severity between mono- and co-infections, there is still a lack of empirical evidence delineating DENV–DENV interactions and immune dysregulation in co-infections, therefore, warranting further rigorous investigations [18,19]. Despite the menacing health effects of dengue co-infections, the exact underlying mechanism remains uncertain.

The complexity of flaviviral co-infections becomes more intricate when it involves different flaviviruses, such as DENV, (ZIKV), WNV, YFV, Saint Louis encephalitis virus (SLEV), and Japanese encephalitis virus (JEV). Among these, co-infections of DENV and ZIKV are particularly noteworthy and frequently reported in prevalent regions, including New Caledonia in 2014 [20], Brazil in 2015, and Colombia in 2016 [21]. The occurrence is primarily due to the overlapping dengue and Zika endemicities. Teng et al. [22] deduced that regions experiencing DENV epidemics are at high risk for ZIKV outbreaks, suggesting a close correlation between the two flaviviruses. The correlation is highly attributable to their shared vectors and similar environmental factors that support their spread and transmission. For example, *Aedes aegypti*, the main mosquito species responsible for transmitting both DENV and ZIKV, thrives in urban environments with high population density and poor sanitation in tropical regions [23]. A more pressing health concern is the high degree of nucleotide similarity (approximately 60%) between ZIKV and DENV. Increasing clinical and experimental evidence points to a substantial level of antibody cross-reactivity between the two viruses. Specifically, the cross-reactivity can lead to antibody-dependent enhancement (ADE), where antibodies developed against dengue can potentially enhance the adversity of Zika. As a result, it further complicates the disease management and public health response [24,25,26]. Apart from becoming a public health burden, ADE is a significant concern in dengue and Zika vaccine development [27]. ADE arises when antibodies produced from a previous infection or vaccination, characterized as either non-neutralizing or suboptimal, aid in the entry of the virus into host cells, leading to increased viral replication and worsening the disease severity [28]. Therefore, a feasible dengue or Zika vaccine must elicit protective immunity to individuals without compromising the risk of severe diseases upon subsequent infections [29].

Co-infections implicating DENV with ZIKV are continuously reported in endemic areas [30,31]. Comparatively, co-infections with other flaviviruses such as JEV and SLEV, on the other hand, have only been occasionally reported, especially in regions where the viruses are co-endemic [32,33]. Additionally, previous studies on the situation found that co-infections lead to either viral interference or enhanced replication, depending on the timing of the number of infections [34]. One of the major challenges in managing flaviviral co-infections is diagnostic inaccuracies as the clinical symptoms of flaviviral infections often overlap. Table 1 highlights the clinical similarities commonly observed in flaviviral infections, implying the need for more accurate differential diagnoses.

Although the WHO 2012 guideline encompassing clinical parameters, haematology, and dengue-specific diagnostics has been acknowledged as a routine diagnostic practice [36], misattributing a flaviviral infection with another due to clinical similarities can lead to the omission of necessary treatments, resulting in poorer outcomes. As a result, further investigations into more specific diagnostic approaches are warranted [37]. 

Table 2 presents a comprehensive summary of flavivirus co-infections in which the strengths and controversies in individual articles are explored.

## 4. Severity and Implications of Co-Infections of DENV Serotypes and with Other Flaviviruses

Lin et al. [38] described the potent mutualistic outcomes of flaviviral co-infections on the viral replication and suggested an enhancement of vector competence [29]. In the arboviral sylvatic cycle, vector competence is defined by whether a mosquito vector can support the exploitation of its circulatory system by the infecting virus [39]. Generally, in the vector, the virus must establish infection in the midgut, produce virions that disseminate to the secondary tissues, and sustain its replication throughout the organ system until newly synthesized progeny reach the salivary glands [40]. DENV-ZIKV co-infected mosquitoes produced significantly greater viral loads than those singly infected, particularly when the co-infection involved DENV-2 [38]. The implications of these findings extend beyond simplistic viral interactions as they highlight the potential for enhanced transmission dynamics within human populations. Given that more than 50 million dengue infections are reported each year, primarily linked to *Aedes* mosquitoes, comprehending the intricacies of flaviviral co-infections is imperative for repurposing public health strategies to alleviate disease outbreaks [41]. Plus, vector competence, particularly the extrinsic incubation period (EIP), is greatly influenced by ecological factors such as temperature and humidity [39,42]. As a result, any changes to these environmental conditions may exacerbate the consequences of simultaneous infections through the enhancement of viral replication, alteration of mosquito feeding frequency, and prolonged longevity of mosquitoes [43,44].

### 4.1. Co-Infections of DENV Serotypes

Although all DENV serotypes share a similar infection mechanism, previous findings indicated that some genotypes are more virulent within a particular serotype, leading to a higher likelihood of severe symptoms. This results in a spectrum of disease severity in the affected population [45]. A co-infection of DENV-1 with DENV-2 is associated with a higher frequency of severe thrombocytopenia on admission and lower nadir platelet counts than mono-infected patients [19]. In turn, warning signs and severe disease manifestations are expectedly elevated in co-infected individuals, such as higher risks for developing pleural effusion and at least one severe dengue manifestation. However, some findings reported otherwise where the overall disease severity among co-infected patients was mild, with only a few severe cases without fatality [19,46,47]. In light of the discrepancies, in 2012, WHO broadened the scope of classic dengue fever and introduced the term “expanded dengue syndrome”. Expanded dengue syndrome can be applied to co-infections as it regularly involves multiple vital organs and leads to severe shock [48,49].

Similar to DENV mono-infections, the severity of co-infections can also be divided into mild and severe clinical presentations [3]. For mild cases, a few studies reported that patients concurrently infected with dengue serotypes recovered without relapse. They initially presented with typical dengue symptoms such as high fever, headache, arthralgia, myalgia, retro-orbital pain, and asthenia, but without any haemorrhagic manifestations [50,51,52]. Upon admission, they demonstrated mildly elevated transaminase levels, a common marker for liver inflammation or damage. More interestingly, most of them experienced primary infections [50,53].

In dengue, progression of the disease into severe forms may be influenced by non-viral factors such as the host’s immune response, genetic factors, and predisposing health conditions. Studies conducted in Malaysia, India, and Brazil have recorded a significantly higher intensity of warning signs (90%) and other severe disease manifestations (15%) among patients with concurrent DENV infections [54,55]. A major fraction of patients exhibited elevated creatinine levels, indicating potential kidney involvement, along with pleural effusion. Furthermore, the frequency of patients with severe thrombocytopenia was greater, highlighting the increased risk of haemorrhage and other blood complications [54,55]. Given the multifactorial drivers, it is critical to include DENV co-infections as part of the routine dengue diagnosis to prevent the occurrence of severe dengue, characterized by severe plasma leakage, haemorrhagic manifestations, and organ impairment at the early stage [3]. Although atypical manifestations of dengue are uncommon, patients with concurrent DENV infections did exhibit hepatic, pulmonary, and renal complications [56]. This indicates that co-infections can exacerbate the severity of the diseases, leading to a broader range of organ involvement and more serious health outcomes. In a nutshell, it implies the critical need for close monitoring and timely management of patients with concurrent infections to prevent severe outcomes and improve patient prognosis [57].

### 4.2. Co-Infections of DENV with Other Flaviviruses

The implications of co-exposure and co-transmission of flaviviruses in terms of epidemiology, pathogenesis, and evolution are not fully understood. Limited clinical data and underdiagnosis of flaviviral co-infections add to the poor understanding of flaviviral co-infections in humans and mosquitoes in natural settings. The capacity of *Aedes aegypti* mosquitoes to harbour and transmit multiple flaviviruses and arboviruses simultaneously has been demonstrated [13]. Moreover, in the long run, flaviviral co-infections may exert a significant influence on the virus evolution, both within the mammalian host and the mosquito vector [13]. In this light, co-infections of flaviviruses are not merely hypothetical; therefore, accurate differential diagnoses for the co-infections, particularly during arboviral outbreaks, are urgently needed [13].

By far, ZIKV-DENV co-infections are most discussed and documented. Generally, Zika patients are mostly asymptomatic (60–80%), however, milder illnesses, which typically subside 4–7 days after the symptom onset, are possibly seen in symptomatic patients [58,59,60]. ZIKV-DENV co-infections can result in similar mild symptoms such as fever, headache, joint pain, weakness, conjunctivitis, diarrhoea, nausea, and muscle pain. Serologically, co-infected individuals showed mild thrombocytopenia and leukopenia [20]. Although causing mostly less severe symptoms, fatal cases were reported in adults and foetuses succumbing to ZIKV-DENV co-infections. Most of the casualties had underlying comorbidities. Their histopathological results revealed tubulointerstitial nephritis, acute tubular necrosis, acute motor axonal polyneuropathy, acute demyelinating polyneuropathy, and pneumonia [61]. Discrepancies were, nonetheless, seen in Singaporean patients co-infected by DENV and ZIKV [31]. Besides the typical symptoms, they also showed gastrointestinal sicknesses and atypical rashes. The variations pose challenges to the disease diagnosis and require healthcare professionals to be highly vigilant when assessing patients for possible arboviral infections. This was evident during the Zika outbreak in Singapore, where some patients displayed isolated symptoms [62]. Overall, arboviral infections, particularly dengue, Zika and Chikungunya are known for poor disease prognosis, and patients usually succumb to severe complications due to misdiagnosis or late treatments [58,59]. 

Co-infections of DENV and JEV are also common in Southeast Asia, particularly during the rainy season when *Aedes albopictus*, *Aedes aegypti* and *Culex tritaeniorhynchus* populations surge significantly [33]. As a neurotropic virus, JEV primarily impacts the central nervous system (CNS). Likewise, systemic inflammation induced by DENV can also affect the CNS, making the disease diagnosis more complex [63,64,65]. The pathogenesis of DENV-JEV co-infection remains unclear, nonetheless, DENV and JEV can enter host cells through shared surface receptors, which might be enhanced by non-neutralizing antibodies, if present, hence more adverse outcomes [66,67,68].

Although less frequently reported, cases involving DENV and SLEV have been documented in endemic areas. For example, Heinen et al. [32] reported SLEV and DENV-4 co-infections among Brazilian patients, including a case of triple infection implicating DENV-1, DENV-4, and SLEV. The patients did not exhibit clinical signs and symptoms when the samples were collected, exemplifying the hurdle of early prevention of acute febrile illnesses in those individuals. However, in a co-infection with DENV-3 and SLEV, haemorrhagic symptoms were present but the disease did not exacerbate [69]. The abovementioned scenarios precisely deliberate the complexity and intricacies of flavivirus co-infections, which can be largely influenced by intrinsic and extrinsic factors such as host immune status, viral interference, timing of infections, and environmental conditions [9,70,71].

## 5. Implications of Better Understanding Flaviviral Co-Infections

### 5.1. Better Disease Diagnosis

Classification of dengue based on clinical symptoms alone is complicated by the circulation of pathogens that cause similar clinical symptoms to DENV infection, such as Chikugunya virus (CHIKV) and ZIKV, which may hamper accurate descriptions of DENV dynamics [64,65]. At present, a series of diagnostic tests have been developed for dengue, aiming at different immunological markers for virus recognition or the host immune reaction (Table 3) [72].

ELISA-based dengue assays have shown improved diagnosis accuracy, nonetheless, are still unable to provide serotype-specific identification, thereby limiting the diagnosis precision [73,74]. For instance, DENV- specific IgM-based detection is only feasible 5–7 days post-infection. To counter the limitation, serological assays targeting DENV NS1 are innovated, offering opportunities for early dengue detection. Although the NS1-based assays generate diagnosis sensitivity of approximately 80–100% and specificity of 100%, it is noteworthy that flaviviruses share high similarities in their NS1 proteins, which eventually affect the overall specificity of the tests [73,75]. For instance, DENV-2 infections consistently show reduced NS1 detection sensitivity across multiple platforms, likely due to epitope variations and viral evolution [76,77]. Furthermore, NS1-based assays are incapable of discerning individual serotypes involved in DENV co-infections [76].The NS1 test sensitivity is also compromised (reduced to only 39–47%) in diagnosing secondary infections due to the presence of pre-existing anti-NS1 antibodies. This phenomenon has been well observed in regions where DENV and ZIKV are co-circulating [78,79]. False positives in NS1-based assays can occur when exposed to artificially high viral titers (>10^7^ pfu/mL) [78]. In this light, combination of NS1 RDT, IgM ELISA, and clinical parameters has been utilized to enhance diagnostic accuracy, which improves the diagnostic sensitivity to 90.3% and specificity to 96.2% [79]. In NS1 RDT, ultrafiltration of serum samples collected at the early stages of infection is performed to improve the test sensitivity by 15–20% [80]. Several emerging multiplex platforms, such as serotype-specific NS1 lateral flow immunoassays (LFIAs), have been proposed for more accurate diagnosis due to their high result concordance with that of RT-PCR without cross-reactivity [81]. Although with high throughputs, key limitations persist in those systems, for instance, high operational costs, hence limited use in areas impacted by flavivirus outbreaks [76]. The NS1 detection usually declines after day 4–5 of illness, emphasizing the need for IgM-based assays for more accurate disease diagnosis [77]. A pooled summary of the diagnostic performance of NS1-based assays is presented in Table 4

As aforementioned, the specificity of DENV NS1 assays is greatly affected by cross-reactivity with other flaviviruses, including WNV, JEV, and YFV, which renders false-positive results, complicating the dengue diagnosis. For instance, patients previously vaccinated or infected with other flaviviruses produce antibodies that can react with DENV antigens, thus resulting in misleading results. In terms of DENV co-infection, the majority of the tests are unable to identify the DENV serotypes, and therefore, an early prognosis of severe dengue caused by the infecting serotype is hardly made [10]. Consequently, it delays the disease management and treatment, which is pivotal to reduce the occurrence of severe complications, including haemorrhages, acute respiratory distress syndrome (ARDS), renal failure, and arthritis [82]. 

The relationship between DENV serotype and disease severity has been demonstrated, for example, the likelihood of developing DHF and SD is purportedly higher in patients infected with DENV-1 than DENV-2 or DENV-3 [83]. In addition, although less pronounced, DENV-1 infection is also linked to the occurrence of conjunctivitis, whilst the symptom is less commonly observed in DENV-2 cases. DENV-2-infected patients, on the other hand, are prone to experiencing joint pain and low platelet counts [83]. Additionally, the adjusted risk ratio (ARR) for dengue with warning signs is 5.94 times higher in DENV-2 infection than that of DENV-1 [76]. Comparing DENV-1 and DENV-4, DENV-1 serotype is more likely to cause hemorrhage, epigastralgia, neuroinvolvement, and thrombocytopenia in patients [84]. DENV-3 and DENV-4 are less commonly linked to severe outcomes even amid the predominance of DENV-4 [85]. The discrepancies in the clinical manifestations and disease severity caused by the DENV serotypes highlight the importance of identifying the infecting DENV serotype so patients can receive timely treatments; in terms of long-term management and follow-up care, emphasis can be given to the possible post-dengue complications that influence the patient’s wellbeing [86].

In Zika, the disease complications primarily affect the CNS, which then leads to Guillain-Barré syndrome (GBS). The association of GBS with Zika was first reported in French Polynesia, in which 98% and 100% of GBS patients had anti-Zika IgM and neutralizing IgG antibodies, respectively [87]. The presence of anti-Zika IgM during the convalescence phase enables an early diagnosis 6 days from the symptom onset. However, given the cross-reactivity of the antibodies with other flaviviruses, the test result has to be interpreted alongside that of a plaque reduction neutralization test (PRNT) and an antibody-based diagnosis [88]. PRNT enhances the sensitivity and specificity of Zika diagnosis to 96.8% and 95.0%, respectively, enabling the differentiation of Zika and its fraternity [89]. Nonetheless, PRNT is not without limitations, its labor-intensive process, high costs, and need for specialized facilities, deter the rapid diagnosis [90,91].

To address the challenges posed by traditional diagnostics especially in minimizing contamination and shortening result turnarounds, automated assays for disease detection, including dengue, have been implemented. These systems are more sensitive and specific than the manual methods because they reduce human errors and comply with the standard procedures. At present, some automated assays allow multiplex diagnosis up to 19 pathogens concurrently [72]. For instance, the VITROS automated test can reach 100% accuracy in estimating viral loads [92]. This can tremendously overcome the relatively low result accuracy in rapid tests [93]. In the context of co-infection, Chao et al. [94] developed a multiplex real-time reverse transcriptase PCR assay to detect and identify eight major flaviviruses in mosquitoes using TaqMan fluorogenic probes. Pabbaraju et al. [95] combined specific probes and primers for DENV, ZIKV and CHIKV, allowing differentiation of the three viruses. The test provided conclusive results in less than an hour. Xu et al. [96] innovated a two-tube multiplex real-time RT-PCR assay for simultaneous detection of ZIKV, CHIKV, DENV, YFV, WNV, and JEV. This assay demonstrated high specificity and sensitivity; hence a reliable diagnosis. Wee et al. [97], on the other hand, resorted to high-resolution targeted proteomics to identify specific flaviviral peptides, enabling precise differentiation of infecting flaviviruses. The above innovations duly exemplify the transition of conventional diagnostic methods to refined molecular diagnostics that incorporate advancements such as NGS and CRISPR-based detection systems [98,99]. NGS is an unbiased tool for pathogen detection, typing, and characterization [100]. Additionally, NGS can detect multiple viral pathogens simultaneously, especially in detecting rare or novel flavivirus clades by generating near-complete genomes for accurate serotyping and genotyping [100]. For example, the addition of pan-flavivirus RT-PCR to Nanopore sequencing has shown sensitive and cost-effective detection of a diversity of flaviviruses in clinical syndromic surveillance [100]. 

In the long term, these developments would not merely improve the preparedness for outbreaks and the assessment of disease frequency but would additionally support the creation of more effective therapeutic strategies and the deployment of vaccines in endemic regions, thereby resulting in a decrease in flavivirus-related morbidity and mortality [101,102].

### 5.2. Improved Disease Management and Treatment

Co-infections of flaviviruses produce unspecific disease symptoms, and disrupt immune response that in turn leads to damaging complications [103,104]. Therefore, identifying the etiological agents in a co-infection is crucial for a few reasons: (i) it prevents the disease progression to severe and life-threatening forms; (ii) it helps curb the occurrence of adverse complications particularly in immunocompromised individuals such as pregnant women so intensive monitoring and counselling can be provided to them in follow-ups; (iii) it ensures better disease surveillance and outbreak controls, and implementation of effective public health interventions. 

**Figure 2:**
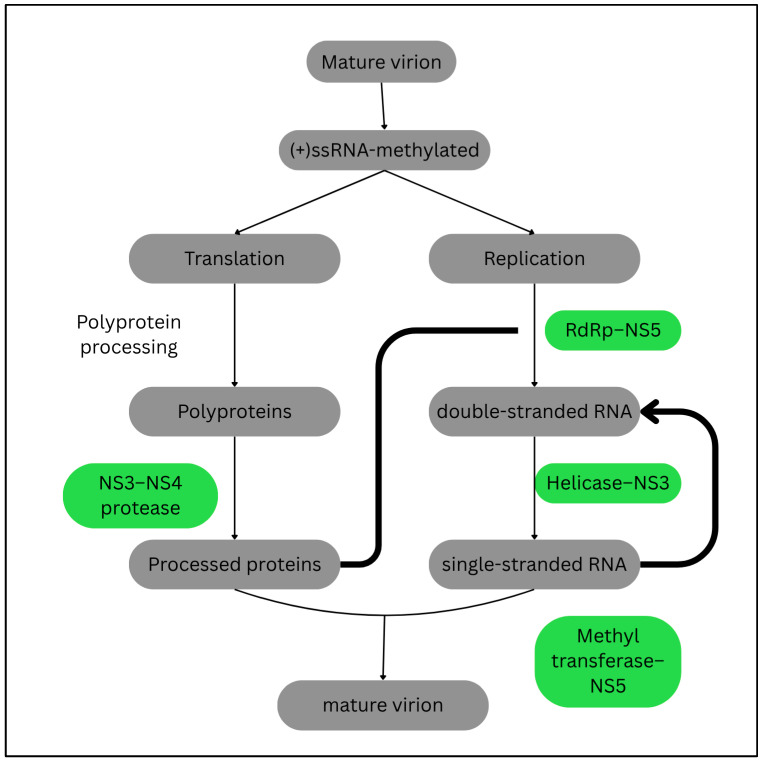
Life cycle of flaviviruses. The key therapeutic targets include the viral genome replicative enzymes (RdRp, helicase, and methyltransferase) and proteases (NS3–NS4 complex). (+)ssRNA indicates positive-sense single-stranded RNA. Adapted from Jayachandran et al. [105].

To achieve those aims, a comprehensive understanding of flaviviral life cycle is crucial. Furthermore, it also provides insights into the specific stages for therapeutic interventions. Figure 2 briefly illustrates the life cycle of flaviviruses and highlights the targeted stages for therapeutic interventions.

The key therapeutic targets of flaviviral life cycle include enzymes involved in the (i) viral genome replication: the viral replicase (NS5), helicase (NS3), methyltransferase (NS5); and (ii) splicing of polyprotein: NS3–NS4 complex [106,107]. Interruptions to these viral molecules undermine the formation of mature virion, thus lowering viral load in the host. Interestingly, similar targets are found in a wide range of flaviviruses, thereby making it possible to invent universal therapeutics for flavivirus infections especially in fighting flaviviral co-infections [106,107].

Effective therapeutics are equally important as control and preventive measures. Bonyah et al. [108] developed a mathematical model to simulate the prevention of dengue fever and Zika in the absence of treatment controls and found that relying purely on control measures did not significantly reduce the occurrence of DENV-ZIKV co-infections. This is further supported by several lines of evidence demonstrating the potential use of antiviral peptides to disrupt the replication of DENV [109]. The inhibitory peptides selectively targeted diverse biological mechanisms, including molecular signalling [110]. Recently, various peptide-based drugs have become available for treating virus infections, for instance, Fuzeon used in the treatment of HIV [110]. Peptide-based inhibitors can disrupt the viral life cycle [111]. They serve as binding partners of viral antigens, thereby blocking the activation of the antigens and viral life cycle [112]. Besides, owing to their small molecular sizes, they can penetrate and cross cell membranes, thereby showing high target specificity and strong binding affinity [113]. Hrobowski et al. [114] synthesised a few peptide candidates to inhibit DENV replication. The peptides disturbed the intramolecular interactions between the stem region and other parts of the class II viral fusion protein, preventing viral fusion and entry. Other peptide molecules such as N-sulfonyl peptide-hybrids exhibited anti-dengue potential through binding to the DENV-2 protease [115]. Interestingly, these peptide candidates were metabolically stable in the rat liver microsomes and pancreatic enzymes, emphasizing their potential as therapeutic anti-dengue agents.

Besides peptide inhibitors, the anti-dengue properties of several synthetic molecular inhibitors were investigated. For example, DV-B-120 and JNJ-A07 that inhibit the DENV NS2B-NS3 protease [116] and disrupt the formation of the NS3–NS4B complex [112], respectively. Inhibitors blocking viral attachment to cellular receptors and entry into host cells are also actively experimented with [109], for instance, N-methylcytisine thio derivatives with high potency to inhibit DENV adsorption to host cell receptors and the activation of NS2B-NS3 protease [117]. Although biologically functional, the stability of the molecular inhibitors remains a major concern due to their small molecular sizes. A few approaches have been adopted to address the problem, such as protein engineering. Specific mutations have been introduced in the protein sequences to solubilize the dimer targeting the DENV E protein [118].

RNA-based approaches, such as RNA silencing and RNA interference (RNAi) have also been attempted to target flavivirus infections like DENV [119,120]. Small interfering RNAs (siRNAs) are used to the complementary viral mRNA sequences, leading to their degradation and preventing the production of viral proteins, hence low viral loads within host cells [121,122]. Interferon therapy can enhance antiviral immunity in hosts [123]. nonetheless, comprehensive investigations are necessary to ascertain their clinical effectiveness and safety profile [124]. By far, type-I interferons (IFNs), including IFN-α and IFN-β, were among the tested interferon candidates that can attenuate ZIKV replication in vitro [123,124]. 

Natural alkaloids such as harringtonine derived from *Cephalotaxus* plant have been proposed for use in treating Zika [125]. In a molecular analysis, harringtone formed interactions with the ZIKV E protein, and therefore was hypothesize to exhibit virucidal and inhibitory effects on ZIKV binding and entry into host cells. Besides ZIKV, harringtonine was also found to exhibit strong virucidal effects against JEV and interfere with multiple stages of the viral life cycle, including binding, entry, replication, and release [125]. 

Co-circulation of flaviviruses in endemic regions emphasizes the need for broad-spectrum antivirals for flaviviruses [126]. Vicenti et al. [127] reported the broad-spectrum anti-flavivirus activities of 2,6-diaminopurine derivatives against flaviviruses, influenza virus, and SARS-CoV-2. Michiaki et al. [128], on the other hand, proposed that 5-aminolevulinic acid (5-ALA) and its derivatives have potential in preventing and treating various flavivirus infections, including dengue, Zika, JE, tick-borne encephalitis, West Nile disease and yellow fever. Valencia et al. [129] recently tested the antiviral effects of two mitogen-activated protein kinase enzymes (MEK) inhibitors, trametinib and selumetinib, and two Src inhibitors, saracatinib and bosutinib, on ZIKV, DENV, and YFV in cell cultures. Trametinib was found to cause 1000-fold reduction in ZIKV and YFV replication and nearly a 100-fold reduction in DENV-2 and DENV-3 replication, suggesting its potential as a broad-spectrum antiviral agent. Those compounds indeed hold promise in improving the treatment regimens for flaviviruses and patient management outcomes, nonetheless, further investigations are needed to validate their efficacy.

### 5.3. Development of Vaccines for Flavivirus Diseases

The ongoing threat posed by flavivirus infections, particularly in endemic and tropical regions, highlights the urgent need for effective vaccines. Over the years, scientific efforts have made remarkable strides, resulting in several licensed vaccines and a wide array of vaccine candidates for clinical use, for instance, several licensed dengue vaccines, including Sanofi’s Dengvaxia, Takeda’s TAK-003, and the NIH-developed TV003/TV005 candidates [130,131,132]. The other successful examples include those targeting JEV, i.e., live attenuated SA14-14-2 and inactivated IXIARO^®^ vaccines [133,134], and yellow fever virus (YFV), namely, YFV-17D live attenuated vaccine [135]. For ZIKV, although no vaccines are currently licensed, several trial candidates, including DNA-based, inactivated, mRNA, and vector-based vaccines, such as Valneva’s VLA1601 and NIH’s DNA vaccine, have progressed to Phase 2/2b [136,137,138]. Meanwhile, there are no licensed vaccines for SLEV and WNV, although veterinary vaccines do exist for WNV [133].

Interestingly, exploration of mRNA vaccines has been largely advocated since the success of the COVID-19 vaccination. mRNA vaccines can induce strong protective immunity and can be duly modified to accommodate multiple target pathogens, as seen in co-infections [139].

Apart from the abovementioned examples, investigations into pan-flavivirus vaccines are increasingly advancing as conventional vaccines primarily focusing on particular flavivirus strains are believed to be insufficient in providing protection against co-infections. One of the approaches involves the development of chimeric vaccines by utilizing insect-specific flavivirus backbones with improved safety profiles and immunization efficacy. Exploration of flaviviral peptides and adjuvants in vaccine compositions to enhance immune response against diverse flavivirus variants are also actively attempted. Rahman et al. [140] investigated the potential of peptide vaccines in stimulating immune protection without triggering allergic or autoimmune reactions.

Advancements in the live-attenuated vaccine technology have vastly improved vaccine efficacy in conferring profound sustainable immunity against various flavivirus diseases [141,142]. Immunoinformatic approaches, on the other hand, enable innovative refinement of live-attenuated vaccines molecularly so newly developed live attenuated vaccine candidates show better safety profiles and stability [143]. A particularly promising molecular strategy involves the manipulation of subgenomic flaviviral RNA (sfRNA) to modify the pathogenicity of the vaccine strains so they can better modulate the host immune responses without compromising the safety of the vaccine [143,144].

Despite the perpetual advancements in vaccine technology, producing flavivirus vaccines faces many challenges beyond production. Hurdles such as antigenic cross-reactivity among flaviviruses exist, which can complicate immune responses and diagnostic interpretations. On top of that, the enhanced disease severity due to ADE, particularly in conjunction to dengue vaccination, is noteworthy [145,146]. Plus, to effectively contain dengue transmission, dengue vaccines ought to contain tetravalent formulations with balanced protection against all four DENV serotypes [145,146].

## 6. Conclusions

Improving the diagnosis of flavivirus diseases, including co-infections, is of utmost importance to provide timely and precise treatment and disease management to patients. It helps tailor effective therapeutic strategies and enhance preventive and control measures for dengue and other flavivirus diseases. This can ultimately prevent the emergence of severe clinical manifestations in co-infections, particularly those caused by ADE. Identifying the infecting flaviviruses and understanding their complex and intricate interactions in co-infections can help develop better diagnostic plans and tools and treatment protocols, ensuring appropriate and timely medical interventions. Therefore, future research should also focus on elucidating the underlying mechanisms involved in the pathogenesis of flavivirus co-infections and the development of broad-spectrum anti-flavivirus agents and vaccines to address the complexities of the diseases and mitigate their public health impacts.

## Figures and Tables

**Table 1:** Comparison of clinical symptoms of dengue, Chikugunya, and Zika (adapted from Ioos et al. [35]).

Disease	Dengue	Chikugunya	Zika
Fever	++++	+++	+++
Myalgia/Arthralgia	+++	++++	++
Oedema in limbs	−	−	++
Maculopapular exanthema	++	++	+++
Retro-orbital pain	++	+	++
Conjunctivitis	−	+	+++
Lymphadenopathy	++	++	+
Hepatomegaly	−	+++	−
Bleeding	+	−	−

“+” indicates the frequency of a symptom seen in patients, “−“ indicates not applicable.

**Table 2:** Summary of flavivirus co-infections: occurrence, clinical relevance, strengths, and controversies.

Category	Specific Aspect	Summary	References	Evidence Strengths/Controversies
1. Occurrence of Co-infection	DENV–DENV co-infection	Co-infections of multiple DENV serotypes have been increasingly reported, particularly during outbreaks in endemic regions. In some areas, the incidences were likely underreported due to limited surveillance and diagnostic sensitivity.	[10,11]	Supported by outbreak data; lacked consistent reporting and large-scale prevalence studies.
	DENV–ZIKV co-infection	Frequently reported due to overlapping endemicity. Documented in New Caledonia (2014), Brazil (2015), and Colombia (2016).	[22,23,24]	Well documented in regional surveillance; supported by ecological overlap.
	Other flavivirus co-infections	Co-infections involving JEV, SLEV, and WNV were less commonly reported, hence their clinical impacts need to be further explored.	[34,35]	Limited data; often incidental reports. Requires more studies.
2. Transmission mechanism, vector, and ecological factors	Vector feeding behaviors	*Aedes* mosquitoes often take multiple blood meals in short intervals, increasing the risk of acquiring more than one virus.	[11,12,13,14]	Strong entomological support; field validation limited.
	Vector competence	*Aedes aegypti* can maintain dual infections due to midgut permissiveness and immune factors. Influenced by virus–virus interactions and host behaviors.	[12,15]	Laboratory models support the mechanism, which is complex in field settings.
	Environmental overlap	Urban environments promote co-transmission due to poor sanitation, high population density, and the presence of shared vector.	[25]	Strong ecological and epidemiological correlation.
3. Clinical relevance and disease severity	Disease outcome	Co-infections may result in higher viremia, leukocyte infection, and cytokine storms, potentially leading to DHF or DSS.	[16,17,18]	Suggested in clinical observations, however, not consistently seen in all cohorts.
	Discrepancies in disease severity	Some studies reported more severe symptoms in co-infections than mono-infections. Others found no significant difference from monoinfection.	[19,20,21]	Conflicting data; clinical heterogeneity and diagnostic variability contribute.
	Timing of infection	Sequential infection timing can lead to either viral interference or enhanced replication.	[36]	Experimental studies supported both outcomes, depending on the infection timing and infecting serotypes.
4. Immunological interactions and diagnosis	Antibody cross-reactivity	Due to ~60% nucleotide similarity between DENV and ZIKV.	[26,27,28]	Well established in serology analyses; significant implications in clinical management.
	Antibody-dependent enhancement (ADE)	Cross-reactive, non-neutralizing antibodies may enhance viral entry, worsening disease severity. Impacts on vaccine development.	[29,30,31]	Strong theoretical and experimental evidence.
	Diagnostic limitations	Overlapping clinical symptoms lead to frequent misdiagnosis. The current WHO guidelines may miss out on co-infection cases.	[34,35,36,37]	Widely acknowledged limitations; highlights urgent need for differential diagnostic tools.

**Table 3:** Commonly used diagnostic tests for dengue detection.

Diagnostic Test	Immunological Target
Polymerase chain reaction	RNA detection
Rapid tests	NS1, IgM, and IgG
Virus isolation	Virus
Immunofluorescence (IF)	Virus, IgM, and IgG
Plaque assay (PA) and fluorescent focus assay (FFA)	Virus titer
Enzyme-linked immunosorbent assay (ELISA)	NS1, IgG, IgM, and IgA
Neutralization test	Neutralizing antibodies (IgG)

Adapted from Herencia (2020) [72].

**Table 4:** Diagnostic performance of NS1-based assays: sensitivity, specificity, and limitations.

Assay Type	Sensitivity Range	Specificity Range	Key Limitations
NS1 ELISA	34–76%	95–100%	Low for DENV-2 and secondary DENV infections.
NS1 RDT	37.8–80%	85–98%	Cross-reactivity at high viral loads.
NS1 + IgM	90.3%	96.2%	Optimal for acute-phase diagnosis.

## Data Availability

Dataset available on request from the authors.
